# Design and test of Kinect-based variable spraying control system for orchards

**DOI:** 10.3389/fpls.2023.1297879

**Published:** 2023-12-21

**Authors:** Xiuyun Xue, Qin Luo, Yihang Ji, Zhaoyong Ma, Jiani Zhu, Zhen Li, Shilei Lyu, Daozong Sun, Shuran Song

**Affiliations:** ^1^ College of Electronic Engineering (College of Artificial Intelligence), South China Agricultural University, Guangzhou, China; ^2^ National Citrus Industry Technical System Machinery Research Office, Guangzhou, China; ^3^ Guangdong Provincial Agricultural Information Monitoring Engineering Technology Research Center, Guangzhou, China; ^4^ Meizhou SCAU-Zhensheng Research Institute for Modern Agricultural Equipment, Meizhou, China

**Keywords:** Kinect sensor, target detection, canopy volume, pulse width modulation, variablerate spraying

## Abstract

Target detection technology and variable-rate spraying technology are key technologies for achieving precise and efficient pesticide application. To address the issues of low efficiency and high working environment requirements in detecting tree information during variable spraying in orchards, this study has designed a variable spraying control system. The system employed a Kinect sensor to real-time detect the canopy volume of citrus trees and adjusted the duty cycle of solenoid valves by pulse width modulation to control the pesticide application. A canopy volume calculation method was proposed, and precision tests for volume detection were conducted, with a maximum relative error of 10.54% compared to manual measurements. A nozzle flow model was designed to determine the spray decision coefficient. When the duty cycle ranged from 30% to 90%, the correlation coefficient of the flow model exceeded 0.95, and the actual flow rate of the system was similar to the theoretical flow rate. Field experiments were conducted to evaluate the spraying effectiveness of the variable spraying control system based on the Kinect sensor. The experimental results indicated that the variable spraying control system demonstrated good consistency between the theoretical spray volume and the actual spray volume. In deposition tests, compared to constant-rate spraying, the droplets under the variable-rate mode based on canopy volume exhibited higher deposition density. Although the amount of droplet deposit and coverage slightly decreased, they still met the requirements for spraying operation quality. Additionally, the variable-rate spray mode achieved the goal of reducing pesticide use, with a maximum pesticide saving rate of 57.14%. This study demonstrates the feasibility of the Kinect sensor in guiding spraying operations and provides a reference for their application in plant protection operations.

## 1 Introduction

Fruit trees are often attacked by pests and diseases during their growth, which not only affects the yield and quality of fruits but also threatens the production efficiency and economic benefits of fruit farmers (Popusoi, 2018). Among many orchard management operations, orchard pest control is the most time-consuming and laborious operation. According to statistics, fruit trees need to be sprayed at least 8 to 15 times per year during the growth period, accounting for about 30% of the total orchard operations (Jiang et al., 2016a). Chemical control is usually carried out through spraying, where spray test is used to disperse the liquid into droplets. These droplets are then applied to the fruit branches and leaves, allowing for chemical control to be achieved (He, 2020). Currently, pesticide application in citrus orchards in China mainly relies on the use of small and medium-sized spray equipment or manual spray poles and spray guns for continuous pesticide application. During pesticide application, the goal is to achieve a rinse-type application mode that thoroughly covers the fruit trees and completely wets the leaves. During the pesticide spraying process, although continuous spraying can achieve high coverage, it overlooks the canopy characteristics of different fruit trees and the variations among them, which often leads to over-spraying, under-spraying and run-off of fruit trees under continuous spraying conditions (Chen et al., 2019; He, 2020; Salcedo et al., 2020).

Variable-rate spraying technology is an intelligent operational method that adjusts the amount of pesticide application based on the characteristics of the target area. As an advanced and efficient orchard pesticide application technology, it can reduce pesticide use by more than 25% compared to continuous spraying (Kang et al., 2011; Stajnko et al., 2012). Variable-rate spraying technology primarily consists of two research directions: (1) Sensor-based detection of fruit tree canopy information, utilizing sensors to detect specific characteristic parameters, such as target fruit tree presence, leaf wall area (LWA), canopy volume (CV), leaf area density, and leaf area index as decision factors for variable-rate spraying (Nørremark et al., 2008). (2) Variable-rate spraying execution system based on different application rate models and flow models, which calculates the amount of application for target fruit trees based on different decision factors, then controls system status including the rotation speed of pumps and the opening and closing of solenoid valves to adjust parameters such as spray pressure, number of nozzles, and liquid flow rate. It ultimately achieves the purpose of on-demand pesticide application (Qiu et al., 2015; Xia, 2016).

The core of variable-rate spraying technology is to accurately obtain target feature information (Zhai et al., 2018). Currently, target feature information detection is primarily achieved through technologies such as laser sensors, infrared sensors, ultrasonic sensors, and machine vision (Balsari et al., 2009; Palleja and Landers, 2015; Berk et al., 2016; Zhang et al., 2018; Comba et al., 2019). The laser sensor detects target canopy structure by measuring the distance from the laser point cloud to the sensor, offering high accuracy and a long detection range. However, the laser sensor system is complex, expensive, and not suitable for environments with high dust, fog, or humidity. As a result, it is challenging to use in practical production. The infrared sensor determines the actual condition of the target by receiving the infrared radiation reflected by the target. It has a short response time, but its detection range is limited and it is highly influenced by lighting conditions (Li et al., 2012). The ultrasonic sensor measures the distance to the target by calculating the time difference between emitting the ultrasonic wave and receiving the echo. It has a farther detection range compared to the infrared sensor. However, its response time is longer, making it unsuitable for real-time detection (Solanelles et al., 2006). The ultrasonic sensor measures the distance to the target by calculating the time difference between emitting the ultrasonic wave and receiving the echo. Although it has a farther detection range compared to the infrared sensor, its response time is longer, making it unsuitable for real-time detection (Solanelles et al., 2006). Machine vision technology can detect the shape of fruit trees and determine the spraying range through image processing techniques. However, monocular vision technology faces challenges in fully eliminating the background, resulting in poor stability. On the other hand, stereo vision technology requires processing a large amount of data, which affects the response speed (Ge et al., 2005; Wang et al., 2019). The Microsoft device Kinect is equipped with both an infrared camera and an RGB camera, enabling it to capture real-time color and depth information within a scene. This combination of machine vision and infrared technology allows the device to leverage the advantages of both.

In the variable-rate spraying execution system, control is achieved through the use of application rate models and nozzle flow models. Researchers have proposed using the decision coefficient to characterize the combined effect of leaf density and leaf wall area on variable-rate spraying, and developing a multi-nozzle flow rate function, which achieves a maximum pesticide savings rate of 68.34% during operations (Xue et al., 2020a). Pulse width modulation (PWM) technology is commonly used to control the spray flow rate when implementing variable spraying (Zhu et al., 2010). When the PWM signal frequency is fixed, there is a good linear relationship between the nozzle flow rate and the PWM signal duty cycle (Fan et al., 2021). At lower PWM signal frequencies (1 ~ 5 Hz), the spray flow rate is less affected by the PWM signal frequency and more influenced by the spray pressure. It also exhibits an approximate proportional relationship with the duty cycle of the PWM signal (Wei et al., 2013). At higher PWM signal frequencies (10 ~ 40 Hz), the impact of different frequencies on the flow rate is also minimal. Although higher frequencies can effectively increase the flow rate adjustment range, they reduce the size of the linear range between flow rate and duty cycle (Li et al., 2016). Under both unregulated and constant pressure spraying conditions, fitting the relationship between the control signal duty cycle and nozzle spray flow rate results in regression equations with determination coefficients greater than 98% (Silva et al., 2018). This indicates that the spray flow models obtained under both spraying conditions have high accuracy and can be used for flow control in the variable-rate spraying execution process.

To systematically investigate the detection methods for canopy volume and spray characteristics of the spraying unit, this study focused on researching target information acquisition and extraction methods using the Kinect sensor based on target detection technology and variable-rate spraying theory, providing theoretical and technical support for real-time detection of canopy parameters. By conducting experimental research and analyzing the application rate decision model for corresponding canopy parameters and the flow model of the corresponding spraying unit, the deposition effects were compared between the constant-rate spraying mode and the CV-based variable-rate spraying mode, to provide theoretical basis and technical support for precise variable-rate pesticide application.

## 2 Materials and methods

### 2.1 Variable-rate spray system

**Figure 1:**
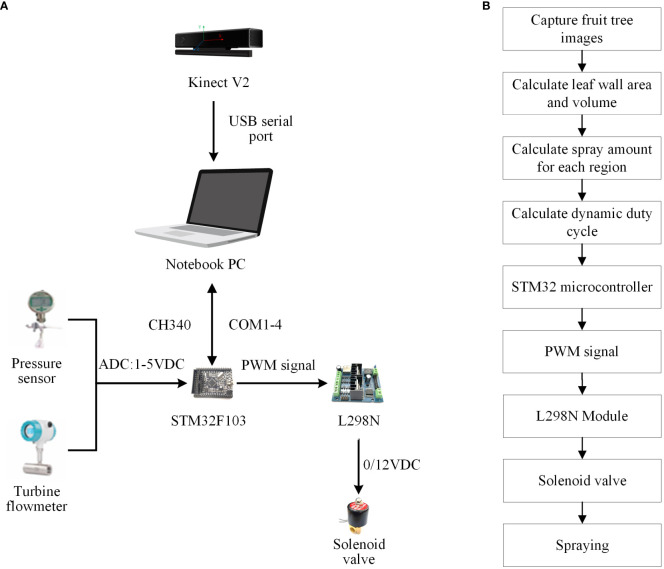
The variable-rate spray system. **(A)** Basic components. **(B)** Control flowchart.

**Figure 2:**
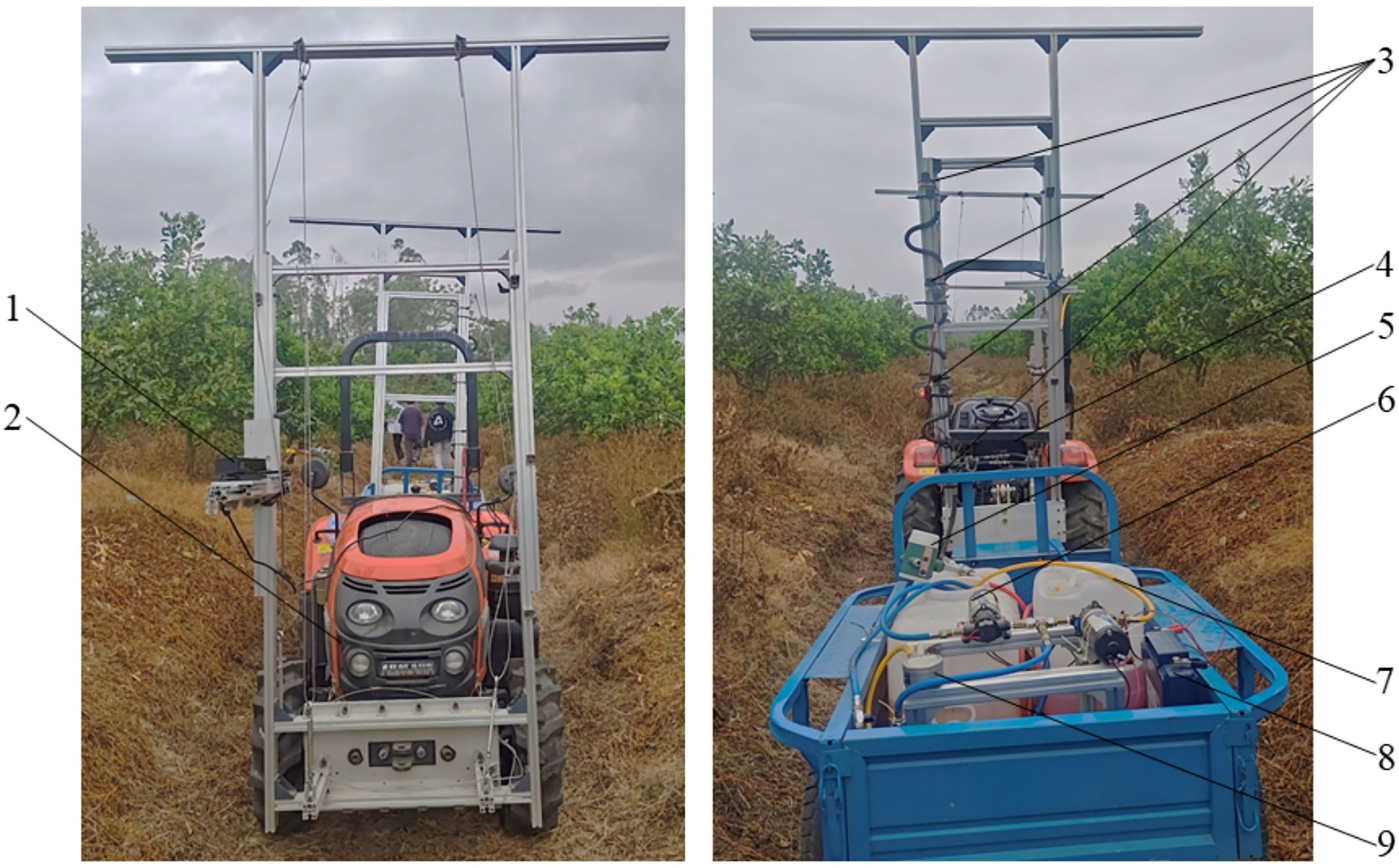
The overall structure of the variable-rate spray system. 1. Kinect sensor, 2. Tractor, 3. Spray unit, 4. Personal computer, 5. Liquid turbine flowmeter, 6. Diaphragam pump, 7. Sprayer tank, 8. DC12V battery, 9. Digital pressure gauge.

The main components of the variable-rate spray system in this research are shown in **Figure 1A**. The system can be divided into target detection unit and variable-spray unit. The target detection unit used a Kinect V2 sensor to collect color information and depth information of the fruit tree canopy in real time and saved it on the laptop. One personal computer was used as an upper computer, responsible for processing the fruit tree information data in real time, determining the spraying scheme based on the application rate decision model, and generating variable spray control instructions, which were then fed back to the spray control module; in the spraying control system, a STM32F103 microcontroller (Minimum system board, STMicroelectronics N.V., Geneva, Switzerland) served as a lower computer and was used to receive and process control command information sent by the upper computer in real time. The spraying equipment was equipped with a standard full cone nozzle JJXP-010-PVDF (H.Ikeuchi&Co, Ltd., Nishi-ku, Japan) and an solenoid valve with a working pressure up to 1 MPa (Delixi Group Co., Ltd., Zhejiang, China). The spraying equipment was mounted at a height of 1.2 meters above the ground, and there were four sets of spray units, each spaced 55 cm apart, as illustrated in **Figure 2**. The chemical liquid was supplied by the diaphragm pump, then it was transported through the pipeline to each solenoid valve, and finally reached each nozzle by the solenoid valve. The overall control process of the variable spray system is to collect the color and depth images of the fruit tree canopy in real time by the Kinect V2 sensor, then transfer the image information to the PC for processing and calculating the leaf wall area and canopy volume, and use the pesticide dosage decision model to calculate the pesticide dosage for the corresponding area, then convert the pesticide dosage into the dynamic duty cycle information for the corresponding area spraying, and finally transfer it to the STM32 microcontroller through serial communication, and output the corresponding PWM duty cycle control command to the four-way drive module L298N, which controls the opening and closing of the corresponding solenoid valve, and then sprays the pesticide through the nozzle. The control flow chart of the variable spray system is shown in **Figure 1B**.

### 2.2 Canopy volume calculation model

In the citrus orchard where field experiments were conducted, the tree row spacing was 4.5 meters, and the tree spacing was 2.5 meters. Based on the imaging principles of the camera and the actual conditions of the citrus orchard, the distance of the Kinect sensor from the center axis of the sprayer, e, was determined to be 0.35 meters, and the installation height from the ground was 1.5 meters (the central position in the direction of the canopy height). For citrus orchards with different planting row spacing and canopy growth conditions, it is available to input the row spacing and adjust the camera installation position so that the sensor’s imaging field of view can adapt to the actual canopy height of the citrus orchard while retaining detection accuracy to the maximum extent. This article only takes the actual situation of the experimental citrus orchard as an example to explain the canopy volume calculation method. The measurement principle of canopy volume is as follows: the sensor uses the canopy’s RGB data and depth data detected within the current field of view as the raw data for calculation. Within the range of the depth matrix currently output by the sensor, the actual height and width of the detection area in the field of view can be calculated in accordance with imaging principles (Yan et al., 2021), as shown in Equation 1.


(1) $$\frac{\text{f}}{\text{R}/2-\text{e}}=\frac{H_{p}}{H_{t}}=\frac{W_{p}}{W_{t}}$$

Where: *f* is the focal length of the sensor, and in this paper, *f* = 3.3*mm*; *H_p_* is the pixel height of the detection area in mm; *H_t_* is the actual height of the detection area in mm; *W_p_* is the pixel width of the detection area in mm; *W_t_* is the actual width of the detection area in mm.

**Figure 3:**
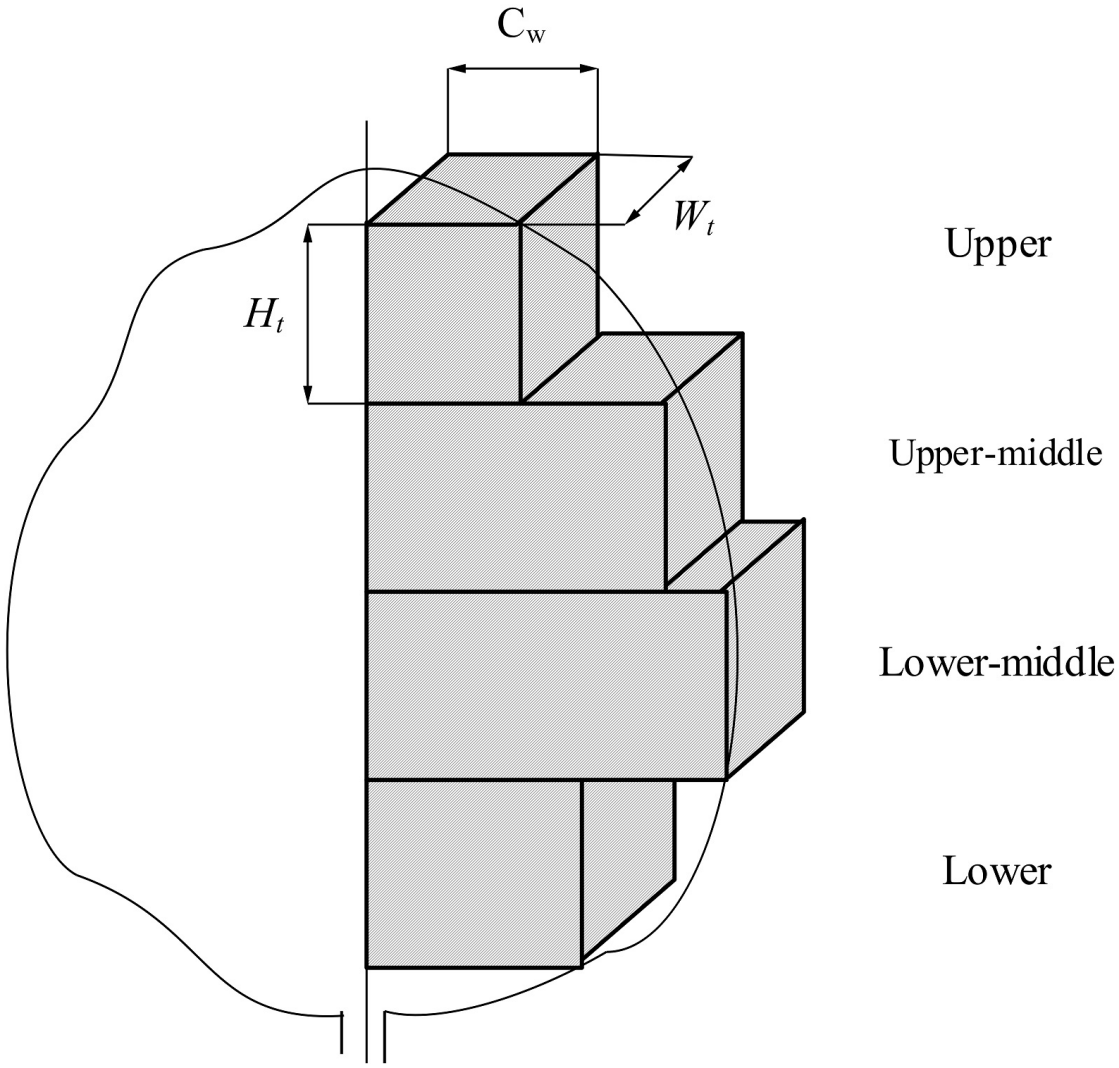
Discretized canopy volume segmentation model.


Considering the spray unit’s spray width and sensor detection accuracy, the 171×424 (width × height) pixel area within the sensor’s central field of view was selected as the spray target area, and the depth values of the 171×424 (width × height) pixel area in the central field of view were selected as the region for target canopy volume. Considering the number of spray units in the actual research, the continuous citrus fruit tree canopy within the sensor’s field of view was discretized into four rectangular volumes: upper, upper-middle, lower-middle and lower. As shown in **Figure 3**, the canopy volume calculation of each unit spray target area was as described in Equations 2-4.

(2) $$C_{V}=S_{LWA}\cdot C_{W}$$

(3) $$S_{LWA}=\frac{H_{t}\cdot W_{t}\cdot N_{LWA}}{N_{all}}$$

(4) $$C_{W}=\frac{R}{2}-\text{e}-\text{d}$$

Where: *C_V_* is the volume of unit spray target canopy in m^3^; *S_LWA_* is the leaf wall area of unit spray target area in m^2^; *C_W_* is the average canopy thickness of unit spray target area in m; *N_LWA_* is the number of pixels in unit spray target canopy; *N_all_* is the number of pixels in unit spray target area; *d* is the depth of unit spray target canopy detected by the sensor in m.

Because the outdoor lighting environment can significantly impact the sensor’s detection accuracy, and based on actual tests, variations in canopy depth detection errors were observed with the binocular camera at different depth detection ranges. By considering the actual depth of the citrus fruit tree canopy and the sensor’s installation position, the canopy detection depth in this study falls within the range of 700 mm to 1900 mm.

**Figure 4:**
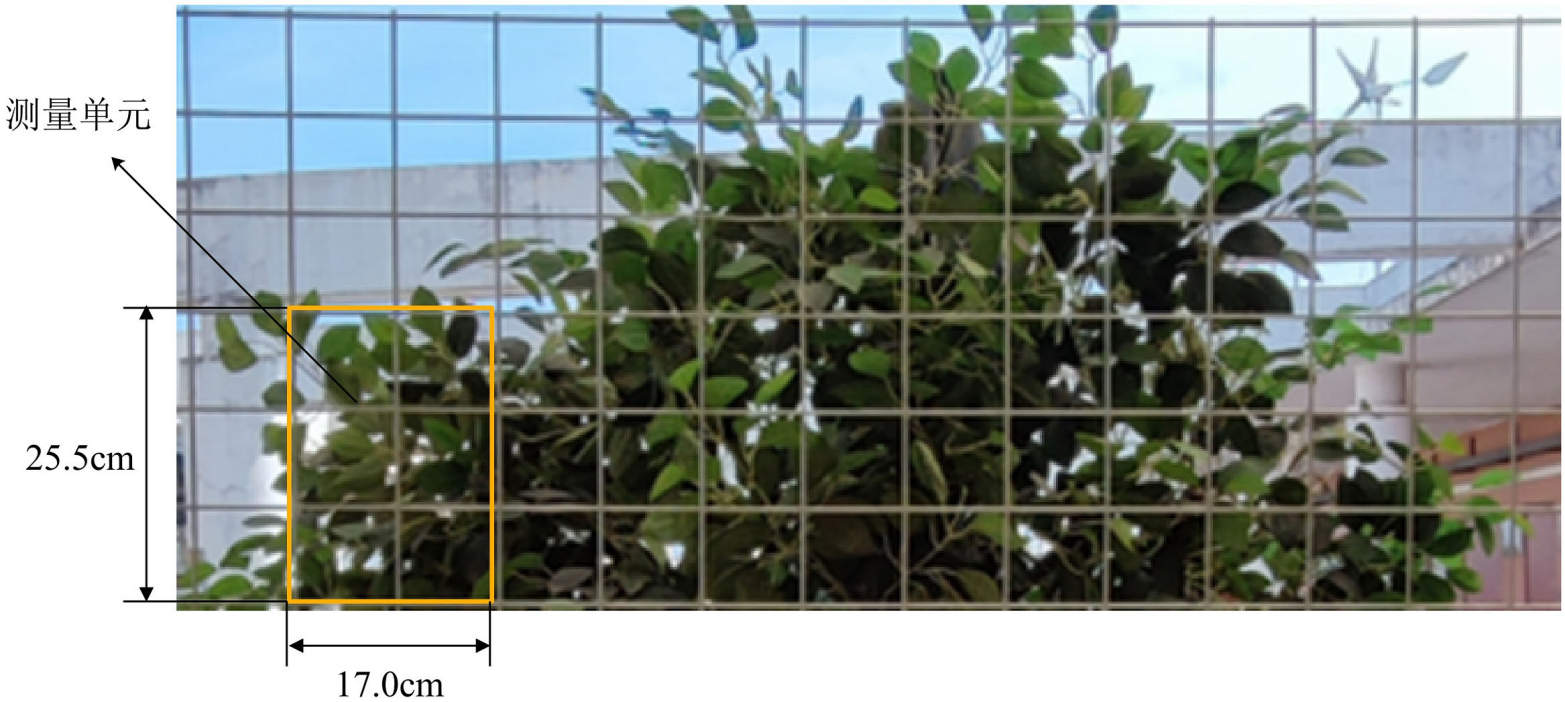
Manual measurement schematic of canopy volume.

To verify the accuracy of the Kinect sensor detection system, five citrus trees at different growth stages were selected for the exploratory test. The test was conducted at a travel speed of 1 m·s^-1^, and each experiment was repeated three times. Additionally, to compare the results obtained from the Kinect sensor detection, the LWA and canopy volume of each citrus tree were manually measured. Referring to the methods employed by other scholars (Rosell Polo et al., 2009; Yan et al., 2021) for manually measuring the area and volume of canopy leaf wall, the canopy was divided from bottom to top into measurement units with a height of 25.5 cm and a width of 17.0 cm in both vertical and horizontal directions. If the height or width at the edges was insufficient, the actual height and width were measured. Then, the LWA of each fruit tree was calculated by summing the area of each unit. When manually measuring the volume, the thickness of each canopy measurement unit was measured three times, and the average value was multiplied by the corresponding unit area to calculate the canopy volume of each measurement unit. Finally, the sum of all unit volumes yielded the canopy volume of the fruit tree. The actual manual measurements are shown in **Figure 4**.

### 2.3 Construction of application rate model

Implementing variable-rate spraying operations with a Kinect sensor primarily involved two components: a decision-making process and an execution process. The application rate model played a role in the decision-making process of variable-rate spraying. It utilized the LWA and canopy volume detected by the Kinect to determine the pesticide application volume in corresponding area. Meanwhile, the nozzle flow model operated during the execution process of variable-rate spraying. With the nozzle flow model, the pesticide application volume was converted into dynamic duty cycle information for the corresponding application area. This information was then transmitted to the STM32 microcontroller through serial communication, and subsequently, relevant PWM duty cycle control instructions were generated to manage the opening and closing of the solenoid valve. This enabled the execution of pesticide application work in the designated area through the nozzle.

The application rate model, designed to make better real-time decisions on the amount of pesticides to be sprayed in the spray area, is shown in Equation 5.


(5) $$q_{flow}=\text{ρv}(\text{a}+\text{b}\cdot \text{K})$$

Where: *q_flow_* is the real-time spray flow in L·min^-1^; *v* is the travel speed in m·s^-1^; *K* is the decision coefficient of the application rate model, K∈[0,1] ; *a* and *b* are constant coefficients of the model, which can be calibrated by the flow model of the nozzle; *ρ* is the coefficient for the spray volume adjustment, taking into account the actual pest control needs in citrus orchards and referencing other scholars’ research (Li et al., 2017; Jiang et al., 2019b), *ρ*=1 was determined.

Fruit trees vary in canopy volumes, which in turn affect the required pesticide quantities for their control. The larger canopy volume of the fruit tree, the corresponding application of pesticide should also increase. The canopy volume of fruit trees is a three-dimensional characteristic that combines canopy area and thickness. To better represent its three-dimensional nature, the decision coefficient for the pesticide application rate model based on parameter canopy volume is denoted as *K_CV_*, and its calculation model is presented in Equation 6.


(6) $$K_{CV}=0.5\times \frac{N_{LWA}}{N_{allI}}+0.5\times \frac{C_{w}}{C_{max}}$$

Where: *C_max_* is the maximum thickness of unit spray target area in m.

The ratio of the average thickness of different volume elements to the maximum thickness of each pixel within that volume element reflects the relative size of the true thickness of the volume element. The decision coefficient *K_CV_* of the CV-based application rate model incorporated information about the LWA and depth within the spray target area, with a 50% weight allocation. This approach better captures the genuine spatial characteristics of fruit tree canopies compared to relying solely on canopy volume for pesticide application (Chen, 2018). It is more flexible and efficient than the use of the original canopy volume values and serves as a guiding factor in rationalizing precise variable-rate pesticide application.

The nozzle flow model operated in the variable-rate spraying execution process and was used for the actual control of spray flow. After obtaining the dynamic spray amount through the application rate model, it was input into the nozzle flow model to derive the relationship between the decision coefficient *K* and the PWM duty cycle. To clarify the flow model of the pesticide application unit and establish the specific relationship between nozzle flow rate and PWM control signal duty cycle, spray flow tests were conducted at spray pressures of 0.3, 0.4, and 0.5 MPa, with measurements taken of the flow conditions at different spray pressures. Taking into account the impact of the PWM signal frequency on nozzle flow rate and the operational frequency of the 2W-025-08 solenoid valve, a control signal frequency of 10 Hz was chosen for flow rate testing. The flowmeter was used to measure the total flow values of four nozzles at different PWM duty cycles. From preliminary tests, it was discovered that when the control signal duty cycle fell below 30%, the solenoid valve coil struggled to maintain stable operation due to difficulties in charging for a short period and discharging for an extended period. In such cases, the nozzle’s spray flow became excessively low, leading to irregular spray operation. Similarly, when the PWM duty cycle exceeded 90%, the electromagnetic force in the valve coil was unable to release quickly, making it challenging to maintain a short-closed and long-open state. In this situation, the nozzle flow was essentially unaffected by changes in the duty cycle. Hence, during the flow rate testing experiments, it was only necessary to measure the flow conditions of the nozzles with duty cycles between 30% and 90%, incrementing by 10%. Due to the high-frequency switching operation of the solenoid valve, there may be fluctuations in the instantaneous flow results from the flowmeter. Therefore, it was essential to measure the total pesticide application quantity over a specific time period and then divide it by the time to obtain the average flow rate during the actual spraying process. In the flow rate measurement experiments of this study, the total spray quantity of four nozzles within a 10-second period was measured using the flowmeter. Each measurement was repeated five times, and the average value was taken as the final measurement result.

### 2.4 Analysis of system response time

Real-time and accurate calculation of dynamic delay time is the guarantee for achieving accurate variable spray. Since there is a horizontal distance between the Kinect sensor and the spray unit, it is necessary to compensate for the delay in the spray command, which ensures that the spray command aligns with the actual spray target area. The time required for delay compensation can be calculated by Equation 7.


(7) $$t_{com}=\frac{L}{v}-t_{sys}$$

Where: *t_com_* is the time required for delay compensation in the spray system in s; *L* is the horizontal distance between the Kinect sensor and the spray unit in m; *v* is the travel speed of the spraying system in m·s^-1^; *t_sys_* is the total response time of the entire variable-rate spray system in s.

According to Equation 7, when $\frac{L}{v}>t_{sys}$ , the system can achieve correspondence between the spray command and the spray area through delay compensation in the software. When $\frac{L}{v}<t_{sys}$ , the correspondence of the target area can only be achieved by adjusting the installation distance between the sensor and the spray unit.

The response time of the variable spray system is mainly composed of four parts, including:

(1) Front-end time *t*_1_: it takes *t*_1_ for Kinect sensors to collect tree canopy information from start to finish;
(2) Data processing time *t*_2_: the time taken after the Kinect sensor has collected data, for the calculation model to compute specific canopy parameters and then the pesticide application decisions to obtain PWM duty cycle information. By setting “start = clock()” in the software program as the time when the canopy information calculation begins, and setting “end = clock()” as the end time when the canopy information is fully converted into duty cycle information, the difference between the two can be calculated as the data processing time *t*_2_.
(3) Communication time between the upper computer and the lower computer, *t*_3_, can be calculated using Equation 8.

(8) $${\text{t}}_{3}=\frac{8\times \text{n}}{\text{B}}$$

Where: *n* is the number of bytes in serial communication; *B* is the baud rate in serial communication and, and *B* is set to 115200; the calculated communication time between the PC and STM32 microcontroller is represented by *t*_3_.

(4) Spray response time *t*_4_: duration starts from when the lower computer receives the duty cycle information until the solenoid valve responds to the nozzle and initiates the spraying process.

**Figure 5:**
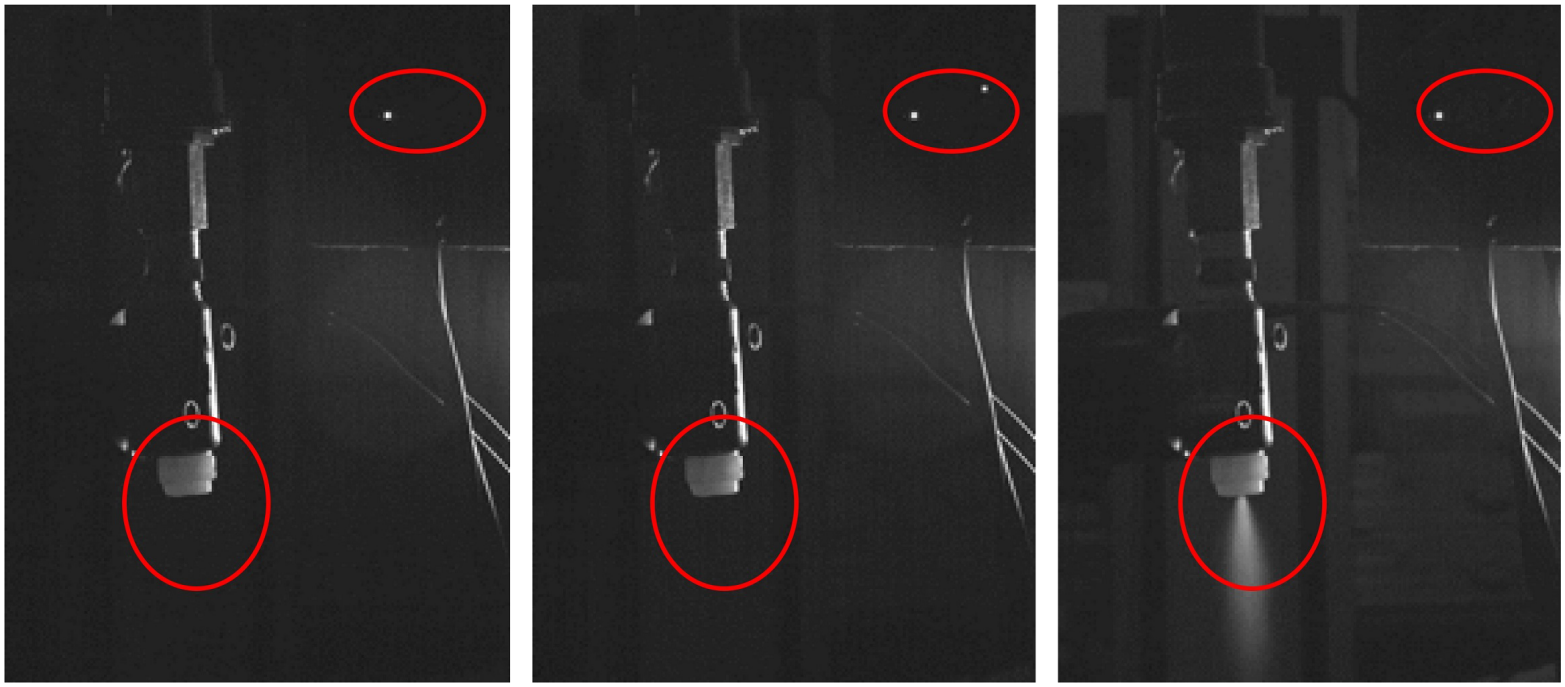
Spraying process captured by high-speed camera.

The spray response time can be calculated by capturing the spray response process using a high-speed camera. In the control program, the LED light was adjusted to reflect the status of serial communication and communication completion. During the high-speed camera capture, set the exposure time to 0.916363 ms and the frame rate to 1056.250 fps. Recorded the timing of the nozzle spray before and after communication with the lower computer, as depicted in **Figure 5**. Then calculated the spray response time *t*_4_.

After calculation, the total system response time *t_sys_* was 203 ms, less than 300 ms. When the travel speed of the spraying system was 1 m·s^-1^, a horizontal distance greater than 500 mm between the spray unit and the Kinect sensor ensured that the system can achieve the matching between the characteristics of the corresponding spray target area and the spray instructions through delay compensation.

### 2.5 System performance verification tests

Due to the hardware response and control capabilities of the variable-rate spray system, which may result in variations between the actual flow rate of the nozzle and the theoretical flow rate, a consistency test between the actual spray flow rate and the target flow rate was conducted under static conditions before proceeding with the spray deposition test, referring to the test methods used by other scholars (Sun et al., 2022). During the test, clean water was used as the medium instead of pesticides, the differences between the nozzles were ignored, and a single nozzle was selected for the flow test. First of the test, the simulated LWA ratio and thickness ratio of the corresponding partition were uniformly set to 0.1, 0.2, 0.3, 0.4, 0.5, 0.6, 0.7, 0.8, 0.9, and 1.0 in the PC upper computer program. The corresponding decision coefficient K values were 0.1, 0.2, 0.3, 0.4, 0.5, 0.6, 0.7, 0.8, 0.9, and 1.0. These values were then sent to the STM32 lower computer for the variable spraying test. In each test, the total spray volume of the nozzle within a 10-second timeframe was measured. The measurement was repeated 5 times, and the average value was calculated. Subsequently, the actual flow rate of the nozzle was determined through conversion based on these measurements.

### 2.6 Field test

Deposition effect is the main indicator to evaluate the performance of a spray system. In order to validate the spray effectiveness of the Kinect sensor-based variable-rate spray system, experiments on constant spray and CV-based variable spray were conducted both indoors and in the field using full cone nozzles and fan-shaped nozzles to analyze the deposition effects.

The main evaluation parameters of deposition effect include droplet deposition density, droplet deposition amount and droplet coverage, calculated by Equations 9–11 respectively. Droplet deposition density refers to the number of droplets per unit area in the target area, measured in droplets·cm^-2^. In plant protection spraying, it has been observed that higher deposition density results in higher efficiency of pesticide, which contributes to effectively reducing pesticide waste (Ahmad et al., 2021). Droplet deposition amount refers to the volume or mass of the chemical liquid per unit area in the target area, typically measured in μL·cm^-2^ or μg·cm^-2^. It directly reflects the quantity of active ingredients of the chemical solution acting on the target and can be used to calculate the pesticide utilization rate. Droplet coverage refers to the ratio between the area covered by droplets on the target surface and the total area of the target. It can be used to measure the coverage of droplets during the spraying process. The larger the coverage rate, the greater the area of droplets attached to the target surface. At the same time, a smaller coefficient of variation for the droplet coverage indicates better penetration of the droplets (Chen et al., 2020).


(9) $$\varepsilon =\frac{A_{1}}{A_{2}}\times 100\%$$

(10) $$\lambda =\frac{n}{A_{2}}$$

Where: *ε* represents the droplet coverage in %; *A*_1_ represents the area covered by droplets in the water-sensitive paper region in cm^2^; *A*_2_ represents the total area of the water-sensitive paper region in cm^2^; *λ* represents the droplet deposition density in droplets/cm²; *n* represents the total number of droplets in the water-sensitive paper region.


(11) $$\gamma =\frac{Ce_{1}\cdot V}{Ce_{2}\cdot S}$$

Where: *γ* represents the deposition volume of droplets per unit area in μL/cm²; *Ce*_1_ represents the concentration of methyl orange in the elution solution in mg/L; *V* represents the volume of distilled water added before elution in μL; *Ce*_2_ represents the concentration of the methyl orange solution used during spraying in μL/cm²; *S* represents the area of the filter paper in cm².

When the droplet coverage exceeds 17%, the overlapping of droplets will cause significant errors in the measurement of droplet size (Wang et al., 2021), thereby affecting the calculation of droplet deposition amount. Hence, this study employed water-sensitive paper and filter paper to measure the droplet deposition density, droplet coverage, and droplet deposition amount, respectively. Water-sensitive papers with dimensions of 110 mm (length) × 35 mm (width), produced by Chongqing Liu Liu Shan Xia Plant Protection Technology Co., Ltd., were chosen for measuring the droplet deposition density and droplet coverage. Circular filter papers with a pore size of 0.22 μm and a diameter of 50 mm, produced by Shanghai Bandaoshiye Co., Ltd., were selected for collecting droplet deposition amount.

**Figure 6:**
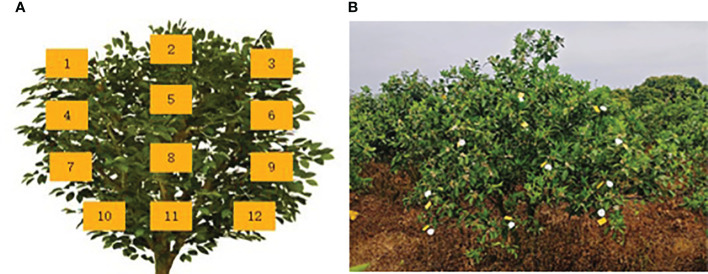
Arrangement of sampling points for deposition test. **(A)** Distribution of sampling points in the fruit tree canopy. **(B)** Distribution of water-sensitive paper and filter paper.

To evaluate the effectiveness of the variable-rate spraying system based on CV and the pesticide saving rate, a spray test was conducted on citrus fruit trees. In the conducted test, methyl orange and distilled water were combined in order to create a solution with a concentration of 0.5 g·L^-1^ of methyl orange, which served as the medium to be used instead of pesticide. The field test was conducted in a citrus orchard at Daju Fruit Industry in Pingtan Town, Huiyang District, Huizhou City, Guangdong Province. The spacing between rows of fruit trees was 4.5 meters, and the spacing between individual trees was 2.5 meters. During the test, the ambient temperature ranged from 21 to 26 °C, the ambient humidity ranged from 46% to 55%, and the ambient wind speed was at level 0 (also known as calm wind, with a speed below 0.2 m·s^-1^). Due to the symmetry of the sensor detection angle, the spray deposition experiment was conducted on a single side of the citrus tree canopy. According to the national industry standard JB/T 9782—2014, the sampling points in the canopy of the fruit tree were arranged as shown in **Figure 6A**. The target tree was divided into four layers vertically: upper, upper- middle, lower- middle, and lower, each of which had three sampling points, numbered from 1 to 12 from left to right and top to bottom. At each sampling point, one water-sensitive paper and one filter paper were placed, as illustrated in **Figure 6B**. To minimize the randomness of the experimental results, each group of the test was repeated three times.

Based on previous tests, the spray test was performed at a spray pressure of 0.4 MPa and a travel speed of 1 m·s^-1^, during which a distance of 30 m was covered each time. The total flow rate values from the flowmeter were recorded before and after the test to calculate the amount of pesticide application. After the test, once the water-sensitive papers had dried, they were sequentially removed along with the filter papers using disposable gloves. The water-sensitive papers and filter papers were then stored separately in sealed bags to prevent moisture damage. Upon returning to the laboratory, the collected water-sensitive papers were scanned at a resolution of 600 dpi using a scanner and saved for further analysis.

**Figure 7:**
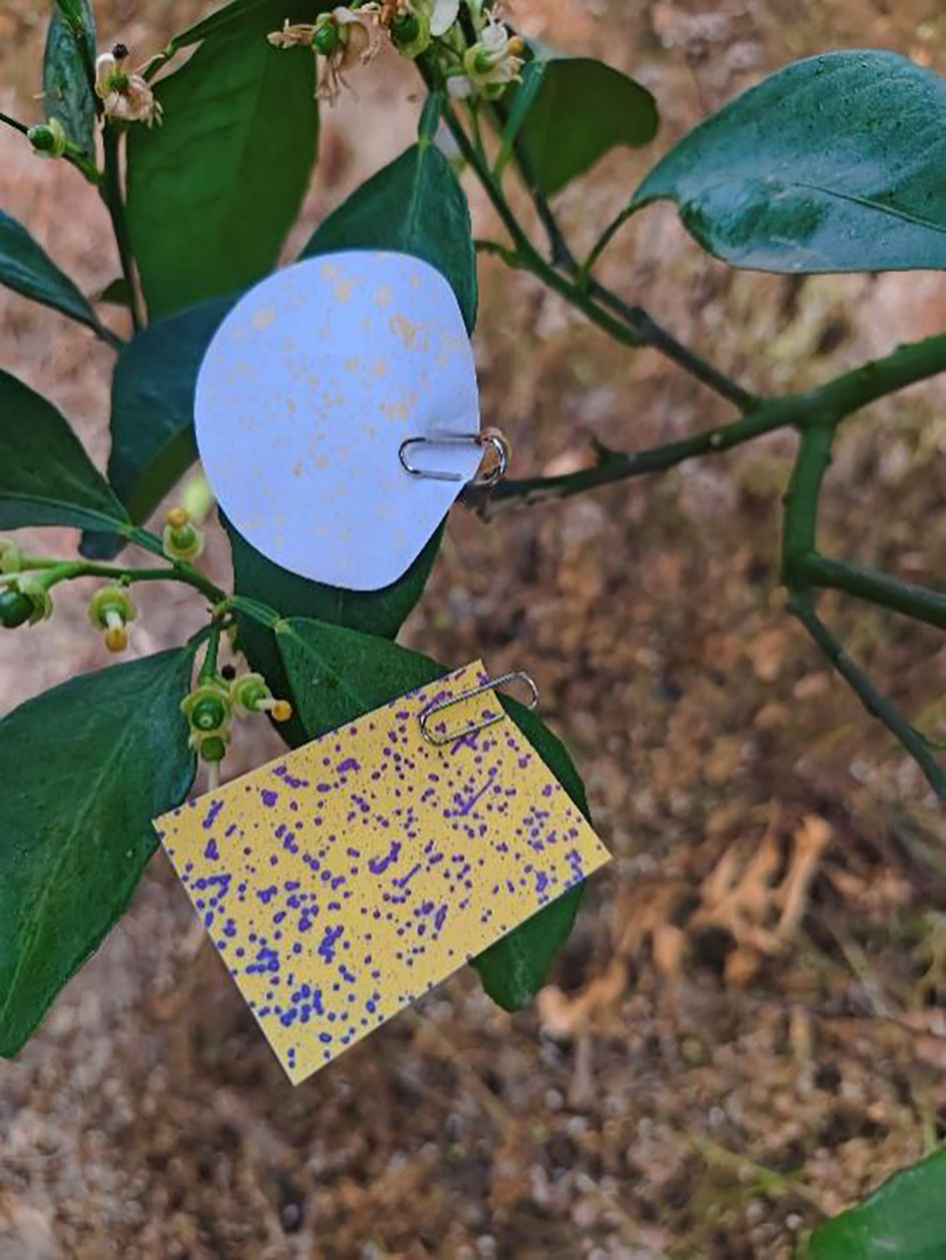
The water-sensitive paper and filter paper after the spray test.

Following the completion of the test, samples of water-sensitive paper and filter paper were obtained, as shown in **Figure 7**. To process the water-sensitive papers, the scanned images were imported into the image processing software Deposit Scan. After configuring the scale, the color images of the water-sensitive paper were converted to 8-bit grayscale. Then, suitable regions and thresholds were selected to process the images, and the parameters of droplet coverage and droplet deposition density were calculated separately using Equations 9 and 10:

To determine the droplet deposition on the filter paper, a UV/Visible spectrophotometer (UV-752, Shanghai Tianpu Analytical Instrument Co., Ltd) was first used to calibrate the concentration-absorbance of the methyl orange solution. Through preliminary calibration tests, a linear regression equation (R²=0.998) was obtained by fitting the concentration-absorbance calibration results, shown as Equation 12.


(12) Abs=0.03461Ce−0.0005

Where: *Abs* is the absorbance value of the measured solution; *Ce* is the concentration of the measured methyl orange solution in mg·L^-1^.

Subsequently, 10 mL of distilled water was added to each filter paper stored in sealed bags. They were then subjected to shaking on an oscillator and elution for 30 minutes. Afterward, 3 mL of eluate was separately measured for absorbance using a spectrophotometer at a wavelength of 465 nm. Finally, the deposition volume of droplets per unit area, which was the droplet deposition amount, was calculated based on Equation 11 (Xue et al., 2022b).

To investigate the uniformity and penetration of droplet deposition in the deposition test, it is common to calculate the coefficient of variation for each parameter. In the deposition test, a smaller coefficient of variation for the distribution of droplet deposition indicates a more uniform deposition, indicating better droplet penetration. The specific calculation of the coefficient of variation is shown in Equations 13-15.


(13) $$\text{CV}=\frac{S}{\bar{X}}\times 100\%$$

Where:


(14) $$\bar{X}=\frac{\sum_{i=1}^{n}X_{i}}{n}$$

(15) $$\text{S}=\sqrt{\frac{\sum_{i=1}^{n}{\left(X_{i}-\bar{X}\right)}^{2}}{\text{n}-1}}$$

Where: *CV* is the coefficient of variation for the sample; *S* is the standard deviation of the sample; $\bar{X}$ is the mean of the sample; *X_i_* is the observed values of the sample; *n* is the number of samples in the dataset.

## 3 Results and discussion

### 3.1 Results and analysis of canopy volume measurement

**Figure 8:**
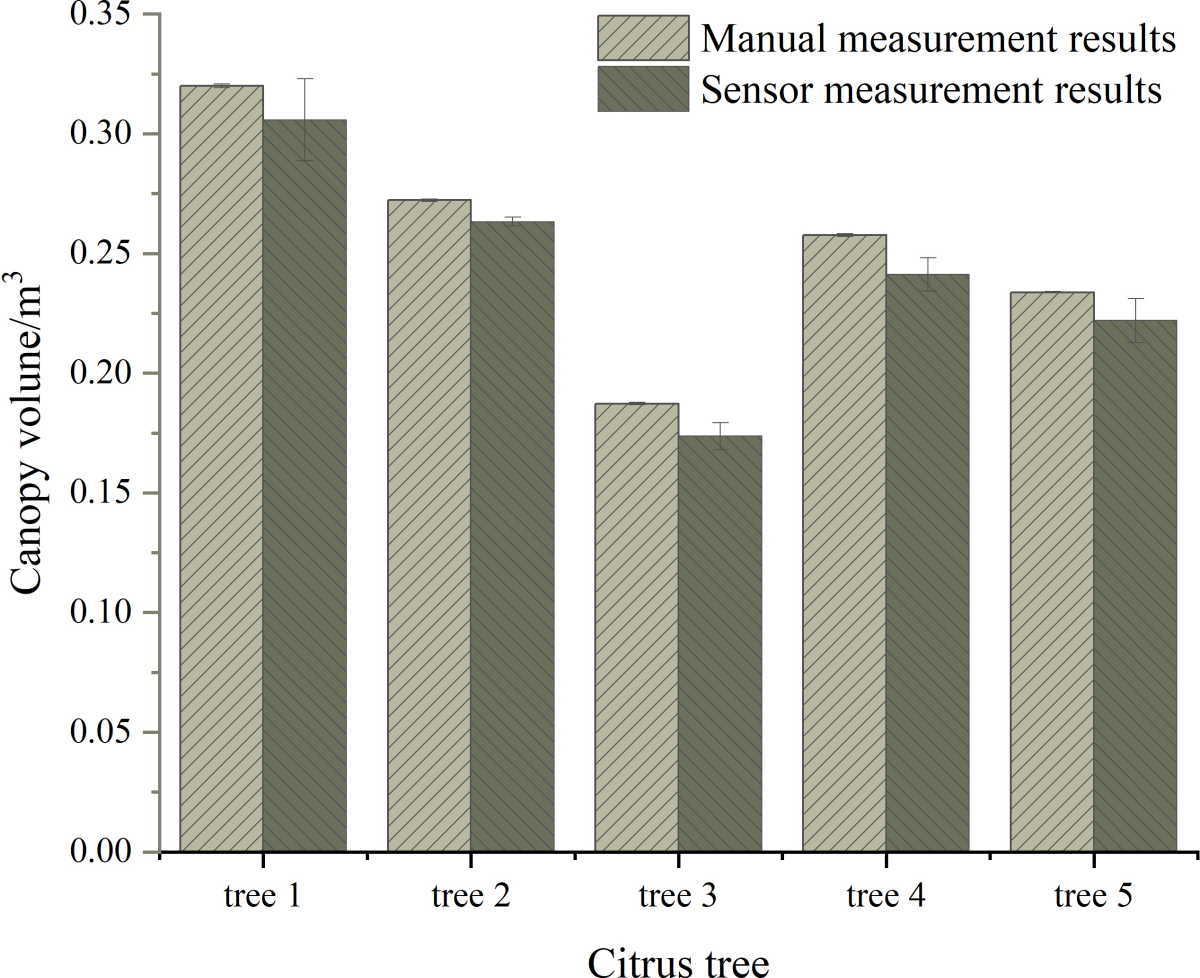
The detection results and relative errors of canopy volume.

The detection results and relative errors of canopy volume for citrus trees at different growth stages are shown in **Figure 8**, with a travel speed of 1 m·s^-1^. The measurements based on the Kinect sensor exhibit ted small deviations, indicating that the detection system had low variability in measuring canopy volume and produced stable results. The calculation model for canopy volume demonstrated its universality. Comparing the sensor measurements to manual measurements, the relative errors were relatively small, ranging from a minimum of 5.98% to a maximum of 10.54%. The Kinect-based citrus tree information detection system and canopy volume calculation model met the accuracy requirements for measuring canopy volume.

### 3.2 The results and analysis of the construction of application rate model

**Figure 9:**
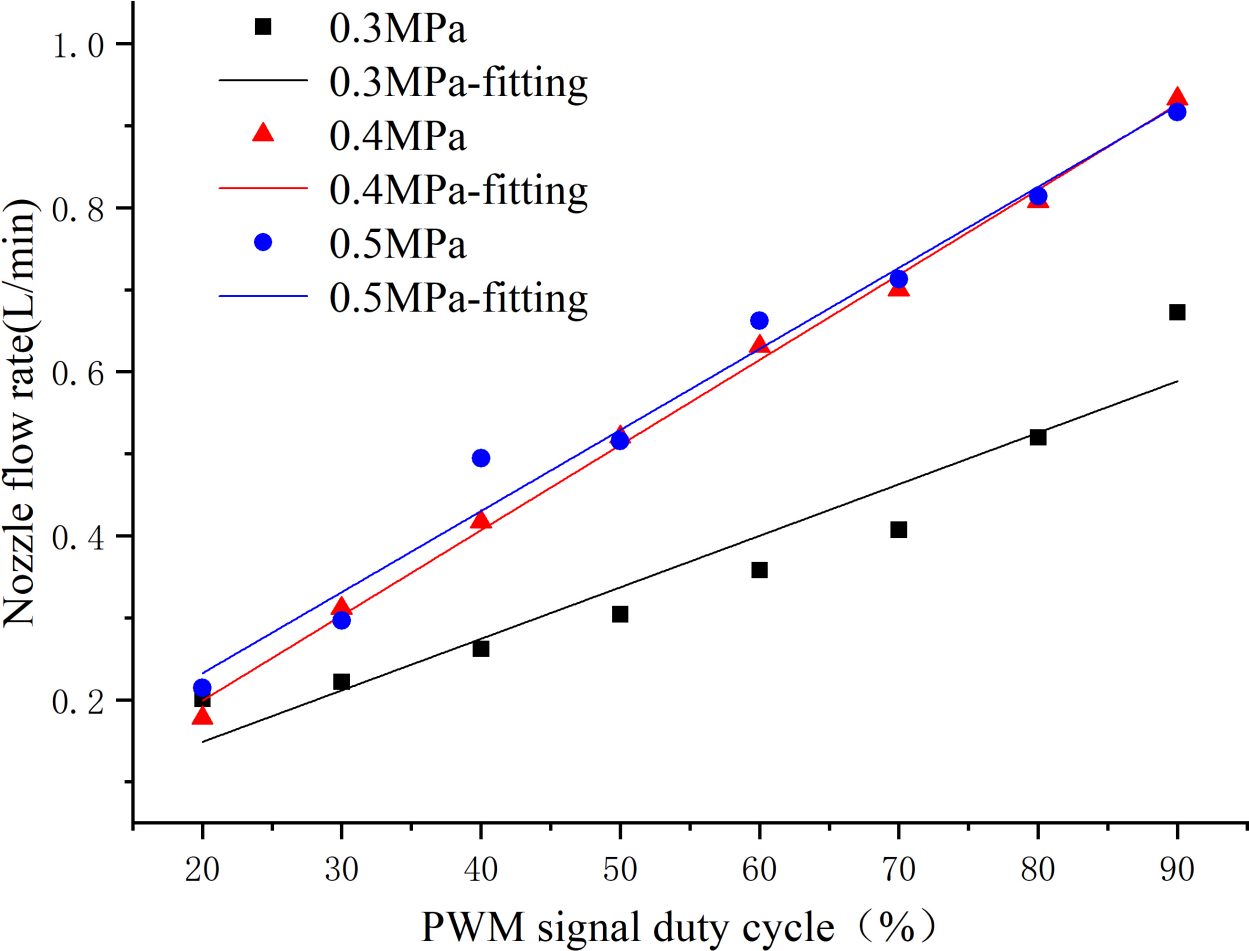
The fitting results of the full cone nozzle flow rate and the PWM signal duty cycle at different spraying pressures.

According to the test design, the average values of multiple sets of test data were calculated, then divided by the spraying time and the number of nozzles to obtain the relationship between the PWM duty cycle and the nozzle flow rate for full cone nozzles and fan-shaped nozzles at spraying pressures of 0.3, 0.4, and 0.5 MPa. During the test, when the PWM control signal duty cycle was less than or equal to 30%, the nozzle spraying was unstable, and the flow rate was too low. But when the duty cycle exceeded 90%, the nozzle flow rate was not significantly affected by the duty cycle. The flow rate data was imported into Origin 2018 software for linear regression analysis. The fitting results of the PWM signal duty cycle and nozzle flow rate at different spraying pressures are shown in **Figure 9**. For spraying pressures of 0.4 and 0.5 MPa, the relationship between the flow rate of the full cone nozzle and the control signal duty cycle was relatively close. However, when the spraying pressure was set to 0.3 MPa, the overall flow rate was smaller due to insufficient pressure.

The fitting results of the spray flow model at three different spraying pressures all had R^2^ values greater than 0.9. Specifically, the R^2^ value obtained from the fitting at 0.4 MPa was 0.997, indicating that the PWM signal duty cycle can explain 99.7% of the variation in nozzle flow rate. Therefore, the spray flow model obtained at this spraying pressure was considered highly reliable. The nozzle flow model for the full cone nozzle at a spraying pressure of 0.4 MPa is shown in Equation 16, where 0.01039 corresponds to the slope of the fitted line at a spray pressure of 0.4 MPa with units of L/(min×%), and 0.00880 corresponds to the intercept of the fitted line with units of L/min.


(16) $$q_{con}=0.01039\text{α}-0.00880$$

Where: *q*_cone_ is the flow rate of the full cone nozzle in L·min^-1^; *α* is the PWM control signal duty cycle in %.

Considering the practical situation of nozzle spraying, when the *α* calculated from the flow model was less than or equal to 30, the response of the solenoid valve became unstable. In this case, the duty cycle of PWM signal *α*_*Duty* controlling the solenoid valve was set to 30. If the *α* calculated was greater than or equal to 90, it indicated that the nozzle flow rate was less affected by the PWM signal duty cycle. Therefore, a duty cycle of 90 was chosen in this scenario. For other situations, a duty cycle of *α* was used. Hence, the specific expression of *α*_*Duty* for the full cone nozzle flow control was given by Equation 17, and the expression of *α*_*Duty* for the fan-shaped nozzle flow rate control was given by Equation 18.


(17) $$\left\{ \begin{array}{c} \alpha \_Duty=30,\;\text{α}\leq 30\\ \alpha \_Duty=\frac{q_{flow}+0.0080}{0.01039},\;30\leq \text{α}\leq 90\\ \alpha \_Duty=90,\;\text{α}\geq 90 \end{array}\right.$$

(18) $$\left\{ \begin{array}{c} \alpha \_Duty=30,\;\text{α}\leq 30\\ \alpha \_Duty=\frac{q_{flow}-0.12131}{0.00906},\;30\leq \text{α}\leq 90\\ \alpha \_Duty=90,\;\text{α}\geq 90 \end{array}\right.$$

By substituting the effective duty cycle range of 30 to 90 into the corresponding flow rate models for the nozzles at 0.4 MPa, the maximum and minimum flow rates for each nozzle at this spraying pressure can be obtained. The results indicated that the flow rate range of the full cone nozzle was slightly greater than that of the fan nozzle, as shown in **Table 1**.

**Table 1:** The extreme values of the flow rates for the full cone nozzle and the fan-shaped nozzle at 0.4 MPa.

Nozzle	Full cone nozzle	Fan-shaped nozzle
Minimum flow rate/L·min^-1^	0.3029	0.3931
Maximum flow rate/L·min^-1^	0.9263	0.9367

To calibrate the coefficients *a* and *b* in Equation 19 while ensuring that the PWM control signal duty cycle ranged from 30% to 90%, the minimum flow rate was taken as the flow rate value when the PWM signal duty cycle was 30%. The corresponding decision coefficient *K* was set to a non-zero extremely small value. Similarly, the maximum flow rate was taken when the PWM signal duty cycle was 90%, and *K* was set to 1. In this case, application rate model for the full cone nozzle is shown in Equation 19.


(19) $$q_{flow\_cone}=\rho v(0.3029\;+\;0.6234\cdot K_{CV})\times 60$$

### 3.3 Results and analysis of performance validation test

**Figure 10:**
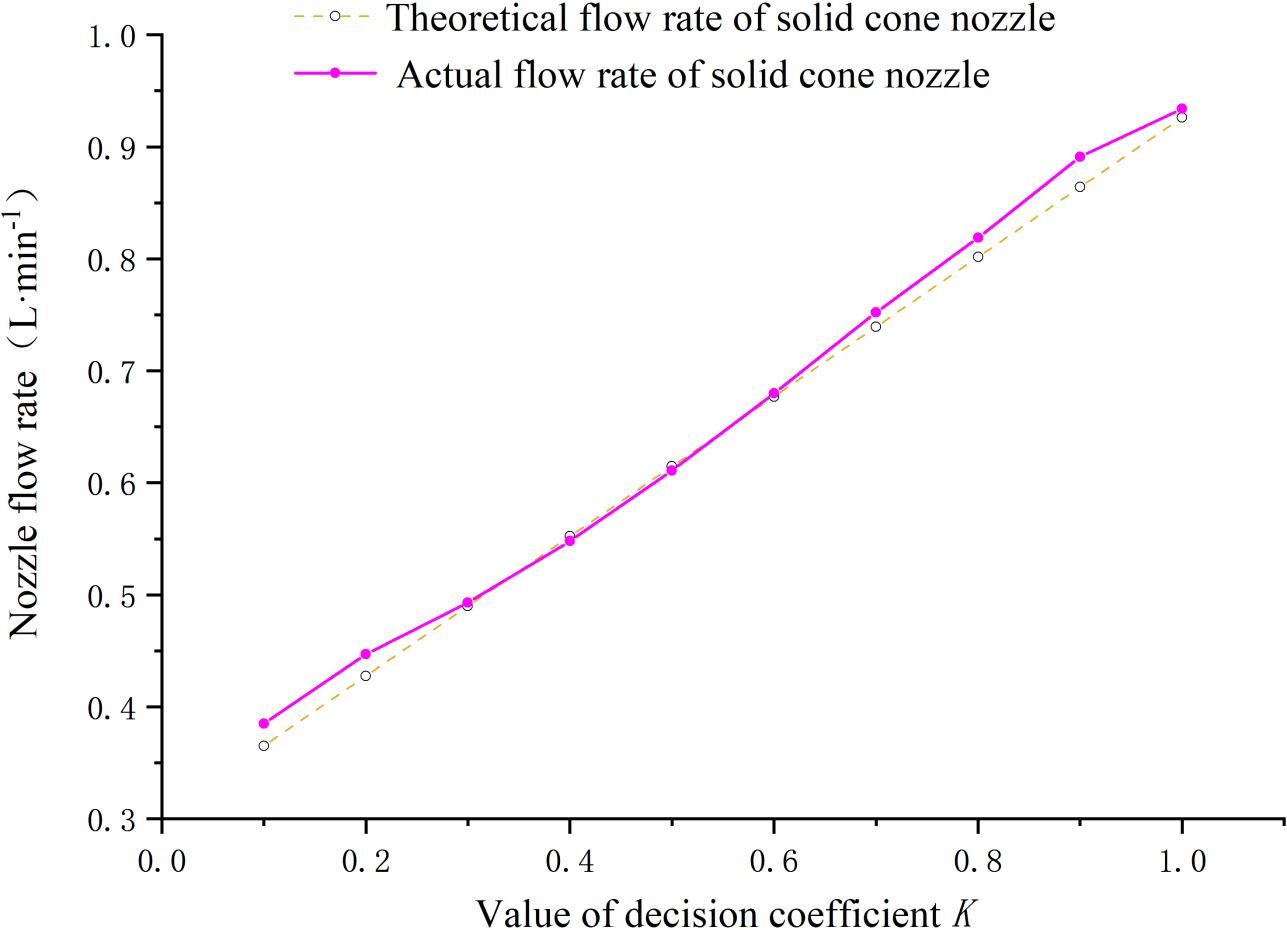
Test results of flow rate consistency for full cone nozzles and fan nozzles.

During the flow rate validation test, the calculated flow rate of the nozzle, obtained by dividing by the spraying time, is shown in **Figure 10**. From the graph, it can be observed that in most cases, the actual flow rate of the nozzle was slightly higher than the theoretical flow rate. However, compared to the theoretical flow rate, the fluctuation range was small. Although it may result in excessive spraying in some canopy areas, it effectively prevented the occurrence of under-spraying or missed spraying in sparse canopy regions.

Furthermore, the graph demonstrates a close proximity between the actual and theoretical flow rates of the nozzle during the spray test. The R^2^ for the decision coefficient of the linear fit exceeded 0.99, indicating strong stability of the control program in the variable-rate spray system, as well as the responsiveness and capability of the hardware equipment to execute the spraying accurately. Moreover, it demonstrates good consistency between the theoretical and actual flow rates for different canopy layers of fruit trees during variable spraying.

### 3.4 Results and analysis field test

In the field test, the average parameter values of droplet deposition under different spraying modes obtained through water-sensitive paper and filter paper treatments are shown in **Table 2**. According to the agricultural standard NYT 650-2013, a spray operation requires a droplet coverage greater than 33% and a droplet deposition density greater than 25 droplets cm^-2^. **Table 1** indicates that the variable-rate spraying based on CV slightly reduced the droplet deposition amount and droplet coverage compared to the constant-rate spraying. However, overall, it still met the quality requirements of spray operations and the droplet deposition density showed an increase. The potential reason for this could be that the constant-rate spraying mode utilized a higher application of the pesticide, causing the atomized droplets to recondense on the foliage of the fruit trees. As a result, the number of droplets decreased. On the other hand, the variable-rate spraying based on CV controlled the spray flow rate. The frequent opening and closing of the solenoid valve created a water hammer effect, resulting in smaller droplet sizes being sprayed. Consequently, more small droplets settles in the canopy of the fruit trees, leading to a higher deposition density.

**Table 2:** Deposition parameters under different spraying modes.

Spraying mode	Full cone nozzle		
	Deposition density/ droplets·cm-2	Deposition amount/μL·cm^-2^	Coverage/%
Constant-rate	42	1.33	43.03
CV-based variate-rate	57	1.12	34.16

#### 3.4.1 Analysis of droplet deposition distribution

**Figure 11:**
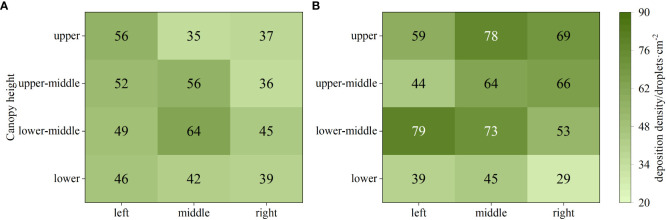
Droplet deposition density under different spraying modes. **(A)** Constant-rate spraying. **(B)** CV-based variable-rate spraying.

The deposition effect of droplets in field test is shown in **Figure 11**, where the deposition density under the variable-rate spraying was significantly higher than that under the constant-rate spraying. This is because during the variable-rate spraying process, the solenoid valve switched its working state frequently, leading to an increase in local pressure at the nozzle of the spray head. As a result, smaller droplets were produced, resulting in a higher deposition density under this mode. Specifically, when conducting CV-based variable-rate spraying, the maximum of droplet deposition density was 79 droplets·cm^-2^, which was a 23.44% increase compared to the constant-rate mode.

**Figure 12:**
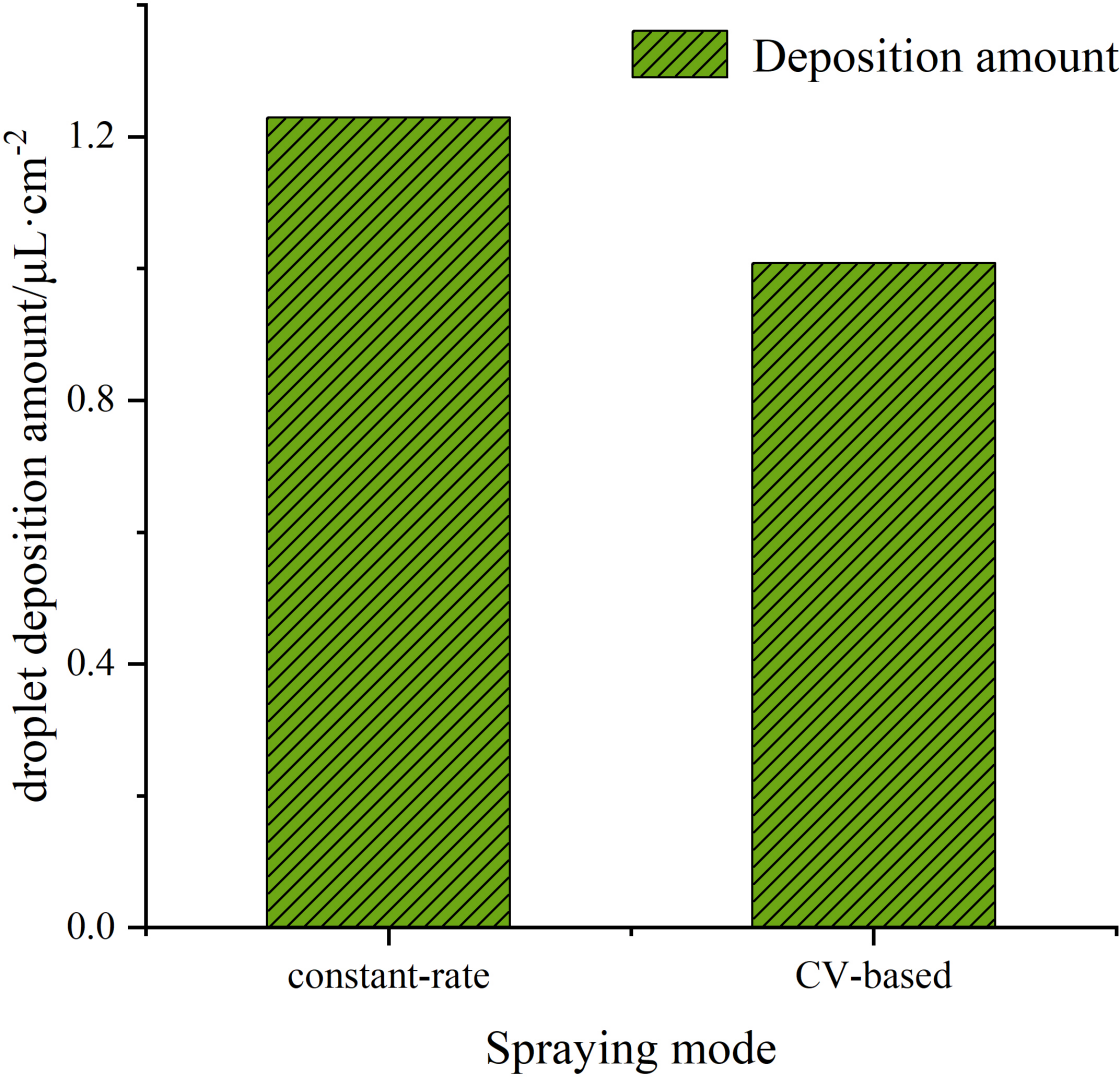
Droplet deposition amount under different spraying mode.

The deposition results of droplets under different spraying modes are shown in **Figure 12**. From the figure, it can be observed that both spraying modes exhibited relatively high levels of droplet deposition. Under the variable-rate mode, the droplet deposition density was higher, but the overall deposition quantity appeared to be slightly lower. This is due to the fact that the droplets produced under the variable-rate mode had smaller diameters, resulting in a smaller volume despite the same number of droplets being present.

#### 3.4.2 Analysis of droplet coverage

**Figure 13:**
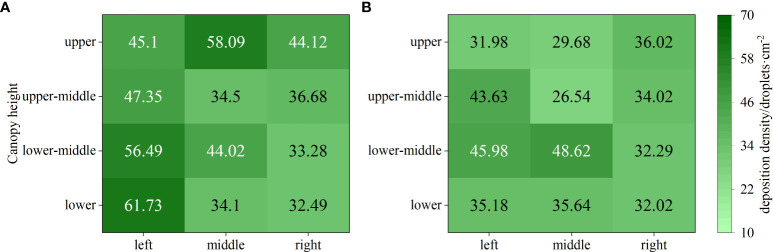
Droplet coverage under different spraying modes. **(A)** Constant-rate spraying. **(B)** CV-based variable-rate spraying.

The droplet coverage results under different spraying modes in the field test are shown in **Figure 13**. The results indicate that in most areas, the droplet coverage under both spraying modes met the quality requirements of the spraying operation. Additionally, the constant-rate spraying exhibited higher droplet coverage compared to the CV-based variable-rate spraying. The droplet coverage under CV-based variable spray was lower, with a decrease of 18.25 percentage points compared to the constant-rate mode.

The smaller the variation coefficient, the stronger the penetrability of the droplets. In this study, the variation coefficient for the constant spray mode was 22.45, while the variation coefficient for the CV-based variable-rate mode was 17.89. From the results, it can be observed that the variation coefficient for droplets under the variable-rate mode was significantly smaller than that of the constant-rate mode. The variation coefficient for the CV-based variable-rate mode was reduced by 20.31 percent compared to the constant-rate mode. Under the variable-rate mode, due to the water hammer effect, droplets exhibited better uniformity and stronger penetrability during the variable-rate spraying process.

#### 3.4.3 Analysis of spray efficiency

In the field deposition test, the application amount for constant-rate spraying was 1.778 L, while the application amount for CV-based variable-rate mode was 0.762 L. Compared to constant-rate mode, the variable-rate mode required a lower application. The CV-based variable-rate mode, using the CV model, achieved a 57.14% reduction in pesticide use. This indicates that, while ensuring spray quality, the CV-based variable-rate mode exhibited a higher spray efficiency in terms of saving pesticide.

## 4 Conclusions

Based on Kinect sensor detection technology and variable spraying techniques, this study constructed a test platform to investigate the canopy volume calculation model for fruit tree canopies, application rate models, spray characteristics of nozzles, and nozzle flow models. The main results and conclusions are as follows:

(1) The study investigated a canopy volume detection model for fruit trees based on the Kinect sensor. By comparing the manually measured canopy parameter values with the results obtained from sensor detection, the canopy volume detection results showed low dispersion and small relative errors. The relative error ranged from 5.98% to 10.54%, verifying the accuracy of the calculation model in the detection system.
(2) The study involved studying and analyzing the use of the decision coefficient K to characterize the canopy characteristics of fruit trees. A corresponding decision-making model for pesticide application rates was established, and the spray characteristics of nozzles and flow models were investigated. Test results demonstrated that under a spray pressure of 0.4 MPa, there was a good linear correlation between nozzle flow rate and PWM control signal duty cycle, with R^2^ greater than 0.95. Based on the fitted nozzle flow model, the flow rate regulation ranges for each nozzle were determined. The constant coefficients in the pesticide application rate model were calibrated, and an expression relating PWM duty cycle to the decision coefficient K was obtained. This expression was used to guide the decision-making and execution stages of variable-rate spraying.
(3) The spray effectiveness of the variable-rate spray system based on the Kinect sensor was tested. The measured theoretical spray volume and actual spray volume showed a high degree of fit, with a decision coefficient R^2^ greater than 0.99, indicating good consistency of the variable-rate spray system. Field test was conducted comparing constant-rate spraying and CV-based variable-rate spraying in terms of droplet deposition density, droplet deposition amount, and droplet coverage. The test results demonstrated that the variable-rate spraying based on Kinect achieved higher droplet deposition density compared to constant-rate spraying. The maximum increase in droplet deposition density reached 28.13%. However, due to the reduction of pesticide dosage, the droplets reaching the target area decreased, and the water hammer effect produced droplets with smaller inertia and more prone to drift, which eventually resulted in the decrease of droplet deposition and coverage, but they all met the quality requirements of spraying operation. Compared to the dosage under constant spraying methods, the drug-saving rate of variable spraying based on CV reached up to 57.14%. In conclusion, the experimental results show that the variable spraying based on CV has a better deposition effect, which can adjust the spray flow rate according to the characteristics of the fruit trees, thereby saving pesticide, improving the efficiency and quality of spraying, and reducing the pollution to the environment and human body. However, our experiment also has some limitations, such as we only consider the leaf wall area and canopy volume of the fruit trees, and do not consider other factors of the fruit trees, such as fruit, flower, etc., which may also affect the deposition and coverage of spraying. In the future, we will further optimize our application rate decision model, consider more factors of the fruit trees, as well as the uniformity and effectiveness of spraying, to find the more suitable parameters for variable spraying.

## Data availability statement

The raw data supporting the conclusions of this article will be made available by the authors, without undue reservation.

## Author contributions

XX: Funding acquisition, Investigation, Methodology, Resources, Supervision, Writing – review & editing. QL: Data curation, Formal analysis, Methodology, Validation, Writing – original draft. YJ: Methodology, Visualization, Writing – original draft. ZM: Validation, Writing – review & editing. JZ: Data curation, Validation, Writing – review & editing. ZL: Conceptualization, Funding acquisition, Resources, Writing – review & editing. SL: Project administration, Writing – review & editing. DS: Supervision, Writing – review & editing. SS: Resources, Writing – review & editing.
